# CHD7 Targets Active Gene Enhancer Elements to Modulate ES Cell-Specific Gene Expression

**DOI:** 10.1371/journal.pgen.1001023

**Published:** 2010-07-15

**Authors:** Michael P. Schnetz, Lusy Handoko, Batool Akhtar-Zaidi, Cynthia F. Bartels, C. Filipe Pereira, Amanda G. Fisher, David J. Adams, Paul Flicek, Gregory E. Crawford, Thomas LaFramboise, Paul Tesar, Chia-Lin Wei, Peter C. Scacheri

**Affiliations:** 1 Department of Genetics, Case Western Reserve University, Cleveland, Ohio, United States of America; 2 Genome Technology and Biology Group, Genome Institute of Singapore, Singapore, Singapore; 3 Lymphocyte Development Group, Medical Research Council Clinical Sciences Centre, Imperial College School of Medicine, Hammersmith Hospital, London, United Kingdom; 4 Wellcome Trust Sanger Institute, Wellcome Trust Genome Campus, Hinxton, Cambridge, United Kingdom; 5 European Bioinformatics Institute, Wellcome Trust Genome Campus, Hinxton, Cambridge, United Kingdom; 6 Institute for Genome Sciences & Policy and Department of Pediatrics, Duke University, Durham, North Carolina, United States of America; 7 Department of Biological Sciences, National University of Singapore, Singapore, Singapore; Medical Research Council Human Genetics Unit, United Kingdom

## Abstract

CHD7 is one of nine members of the chromodomain helicase DNA–binding domain family of ATP–dependent chromatin remodeling enzymes found in mammalian cells. De novo mutation of *CHD7* is a major cause of CHARGE syndrome, a genetic condition characterized by multiple congenital anomalies. To gain insights to the function of CHD7, we used the technique of chromatin immunoprecipitation followed by massively parallel DNA sequencing (ChIP–Seq) to map CHD7 sites in mouse ES cells. We identified 10,483 sites on chromatin bound by CHD7 at high confidence. Most of the CHD7 sites show features of gene enhancer elements. Specifically, CHD7 sites are predominantly located distal to transcription start sites, contain high levels of H3K4 mono-methylation, found within open chromatin that is hypersensitive to DNase I digestion, and correlate with ES cell-specific gene expression. Moreover, CHD7 co-localizes with P300, a known enhancer-binding protein and strong predictor of enhancer activity. Correlations with 18 other factors mapped by ChIP–seq in mouse ES cells indicate that CHD7 also co-localizes with ES cell master regulators OCT4, SOX2, and NANOG. Correlations between CHD7 sites and global gene expression profiles obtained from *Chd7* ^+/+^, *Chd7* ^+/−^, and *Chd7* ^−/−^ ES cells indicate that CHD7 functions at enhancers as a transcriptional rheostat to modulate, or fine-tune the expression levels of ES–specific genes. CHD7 can modulate genes in either the positive or negative direction, although negative regulation appears to be the more direct effect of CHD7 binding. These data indicate that enhancer-binding proteins can limit gene expression and are not necessarily co-activators. Although ES cells are not likely to be affected in CHARGE syndrome, we propose that enhancer-mediated gene dysregulation contributes to disease pathogenesis and that the critical CHD7 target genes may be subject to positive or negative regulation.

## Author Summary

The gene encoding chromodomain helicase DNA–binding protein 7 (*CHD7*) is required for normal mammalian development. In humans, genetic mutations in *CHD7* lead to CHARGE syndrome, a disorder characterized by multiple birth defects. In previous studies, CHD7 was shown to localize to the cell nucleus and bind to specific sites on chromatin. However, the genome-wide distribution of CHD7 on chromatin and its function are not known. Here, we identified 10,483 sites on chromatin bound by CHD7 in mouse embryonic stem cells. Many of these sites are gene enhancer elements suspected to be involved in turning on genes. We show CHD7 functions at these loci to fine-tune the levels of genes that are specifically expressed in mouse ES cells. This modulation is mediated through several proteins that bind together with CHD7 at enhancer elements and can occur in either direction. These findings suggest CHARGE syndrome is the result of key genes that are improperly expressed during development. These key genes are currently unknown but are likely to be tissue-specific and may be upregulated or downregulated in response to CHD7 mutation.

## Introduction

CHD7 (NM_017780) is a member of the chromodomain helicase DNA binding domain family of ATP-dependent chromatin remodeling enzymes. *De novo* mutation of *CHD7* is a major cause of CHARGE syndrome (OMIM 214800), a genetic condition characterized by multiple congenital anomalies [1]. *CHD7* mutations have also been reported in patients diagnosed with diseases that have significant clinical overlap with CHARGE syndrome, including Kallmann syndrome (OMIM 147950) [2]–[4], Omenn-like syndrome (OMIM 603554) [5], and 22q11.2 deletion syndromes [6]. Haploinsufficiency is the proposed mechanism of disease pathogenesis, because most *CHD7* mutations are nonsense and frameshift predicted to be loss of function [7]. Studies in mice support the haploinsufficiency model. Mice that are homozygous for either nonsense or frameshift mutations in *Chd7* (NM_001081417) die around embryonic day 10.5, while heterozygous *Chd7* mutants are viable and develop many of the features observed in CHARGE syndrome [8]. These studies point to a critical role for CHD7 in development, but that role is currently unknown.

CHD7 is a nuclear protein that contains tandem N-terminal chromodomains that mediate binding to methylated histones <@?show=[to]?>[9], a central SNF2-like ATPase/helicase domain predicted to mediate chromatin remodeling, a histone/DNA-binding SANT domain, and two C-terminal BRK domains of unknown function. Expression is widespread and high early in development, with progressive restriction to CHARGE-relevant tissues [8], [10], [11]. It is not known whether CHD7 binds directly to DNA, but a role in transcription has been proposed based on homology to other proteins within the nine member CHD superfamily [12]. Consistent with this notion, CHD7 is homologous to *Drosophila melanogaster Kismet* (NM_078717), a trithorax family member proposed to promote early transcriptional elongation [13], [14].

Structural determinants within the tandem chromodomains of CHD7 are predicted to mediate docking of CHD7 to methylated lysine 4 of histone H3 (H3K4me) [15]. Consistent with this prediction, we recently showed through ChIP-chip studies that the distribution of CHD7 correlates with all three methylated forms of H3K4, with the majority of CHD7 sites overlapping mono- and di-methylated H3K4 (H3K4me1/2) located at regions distal to transcription start sites [9]. Interestingly, the distal CHD7 sites show features of gene enhancer elements [16], [17], i.e., in addition to containing high levels of H3K4me1/2, distal CHD7 sites are cell type specific and contained within “open” chromatin that is hypersensitive to DNase I digestion (DNase HS). Moreover, three out of six CHD7 binding sites functioned as enhancers when tested in luciferase reporter assays. These data raise the possibility that CHD7 is an enhancer-binding protein. However, because these studies were limited to 1% of the genome, only a small number of sites targeted by CHD7 were examined. Furthermore, the relationship between CHD7 binding and cell-type specific patterns of gene expression could not be adequately addressed. Whether or not CHD7 directly functions to regulate transcription was not assessed and remains unknown.

As a first step to investigate the function of CHD7, we used the technique of chromatin immunoprecipitation followed by massively parallel DNA sequencing (ChIP-Seq) [18] to map CHD7 sites in mouse ES cells, representing the earliest precursor to all tissues affected in CHARGE syndrome. By correlating the location of CHD7 sites to histone modifications, gene expression, numerous transcription factors and other publicly available datasets, we show that CHD7 localizes predominantly to enhancer elements. Correlations between CHD7 binding sites and global gene expression profiles from *Chd7* wildtype, heterozygous, and null ES cells indicate that CHD7 functions to modulate, or fine-tune, cell type-specific gene expression. This study establishes CHD7 as a transcriptional regulator, highlights a novel mechanism of enhancer-mediated regulation, and implies that the multiple anomalies in CHARGE syndrome result from dysregulated expression of tissue-specific genes.

## Results

### Characterization of genome-wide CHD7 occupancy using ChIP–Seq analysis

**Figure 1:**
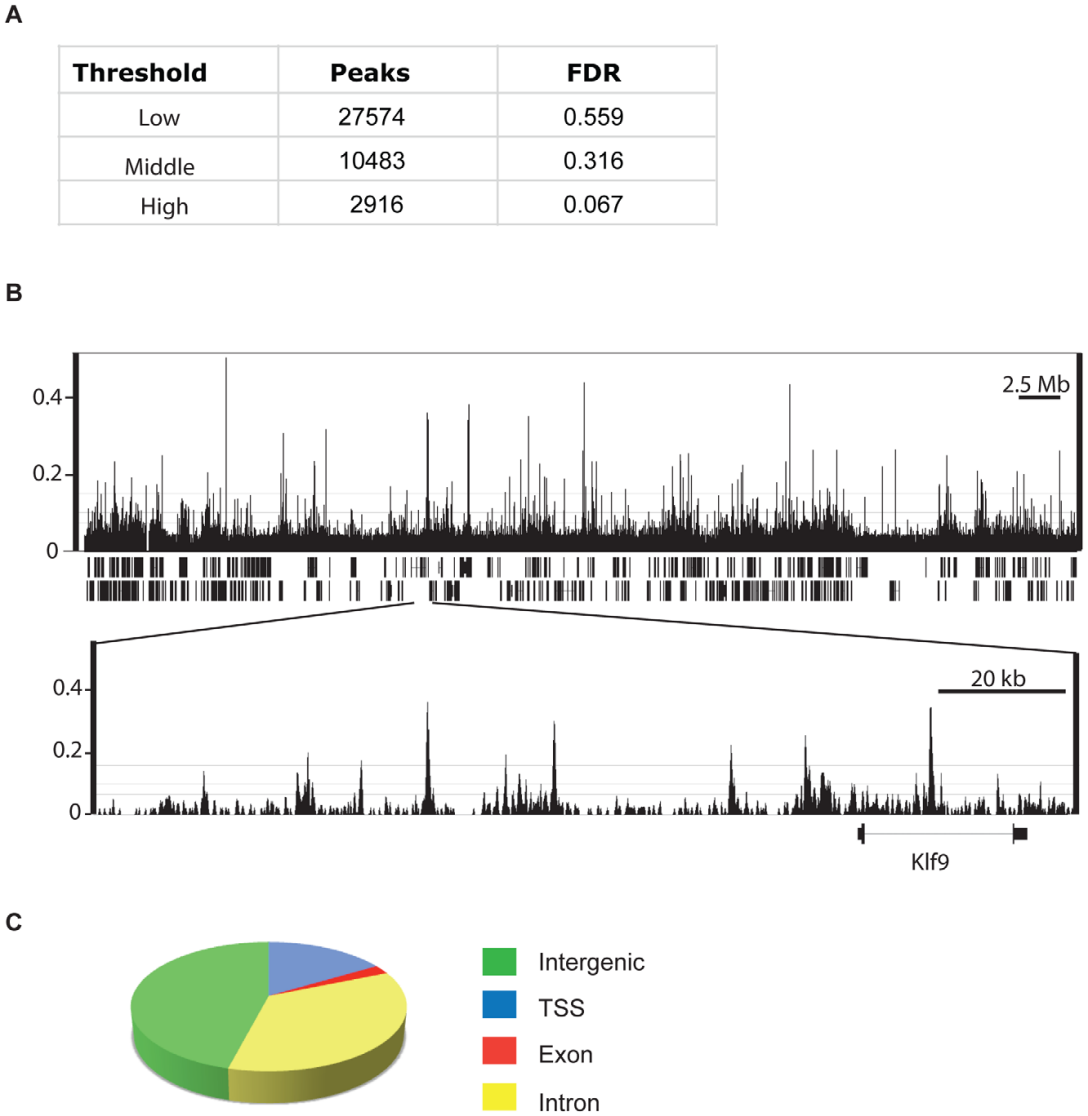
CHD7 ChIP–Seq analysis in mouse ES cells. (A) Total number of ChIP-Seq peaks representing genome-wide CHD7 binding sites at three thresholds and corresponding false-discovery rates (FDR). (B) Integrated Genome Browser view of mapped sequence tags from CHD7 ChIP-Seq analyses with low, medium, and high thresholds shown as horizontal lines. (top panel) Data from mouse chromosome 19. (bottom panel) Zoomed view of individual CHD7 ChIP-seq peaks. (C) Pie chart depicting the location of CHD7 binding sites relative to known genes (mm8 assembly).

We mapped the distribution of CHD7 on chromatin in mouse ES cells using ChIP-Seq. We detected 27574, 10483, and 2916 CHD7 binding sites at low, middle, and high confidence thresholds, respectively (Figure 1A). A representative example of the ChIP-seq data is shown in Figure 1B. False discovery rates (FDR) were calculated by comparing CHD7 sites identified by ChIP-seq to those identified by ChIP-chip on 1% of the mouse genome [9]. These ChIP experiments are biological replicates, and therefore, FDRs may reflect some degree of biological variation or platform-specific differences, rather than true false positives. Nevertheless, at the lowest threshold, 44% of the CHD7 peaks identified by ChIP-seq were also identified by ChIP-chip. These percentages increase to 68% and 93% at the middle and high thresholds, respectively.

**Figure 2:**
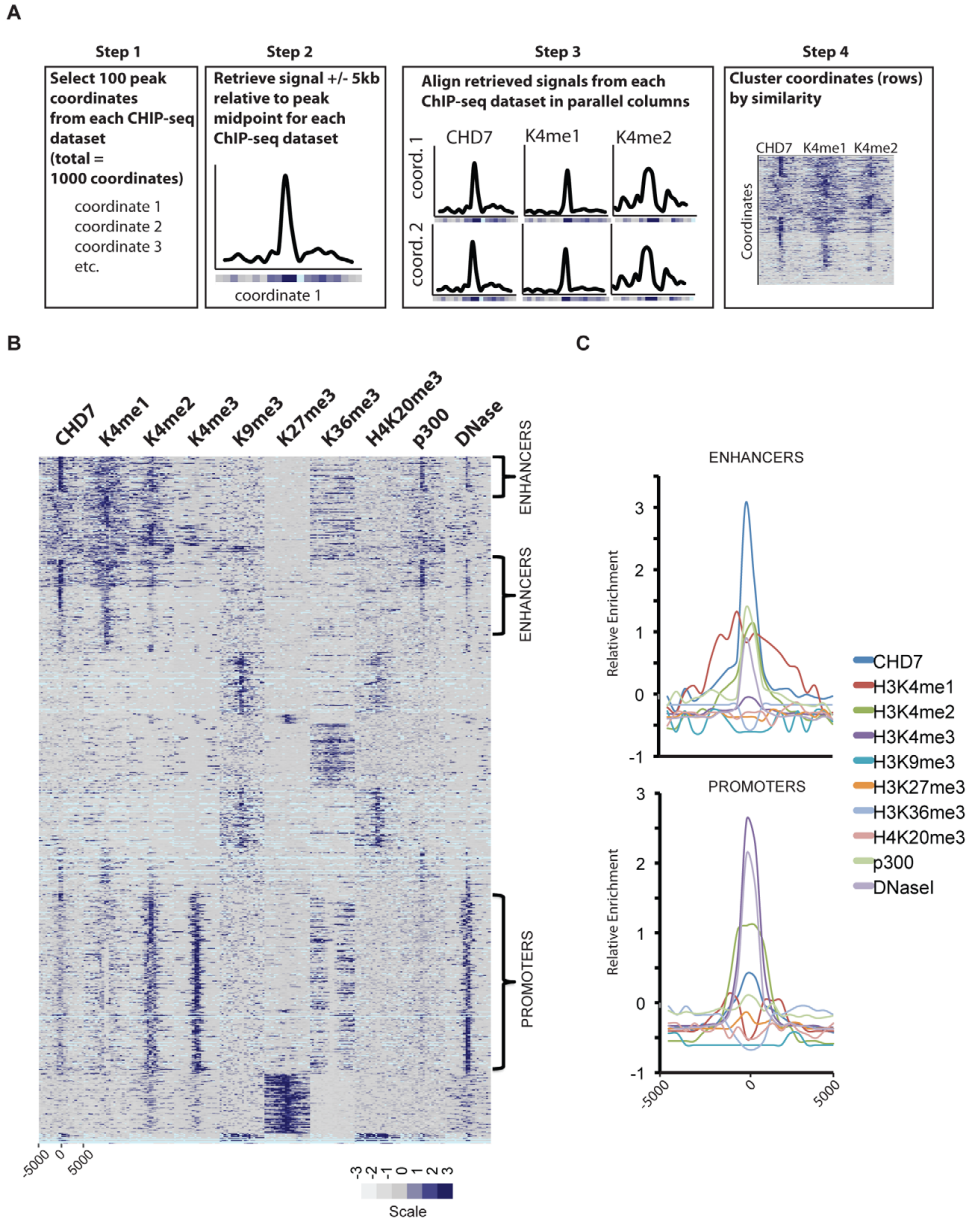
CHD7 localizes to enhancers and promoters. (A) Strategy for characterizing CHD7 binding sites (see Materials and Methods). (B) CHD7 binds to sites containing the characteristics of gene promoters and enhancers. The scale corresponds to relative signal intensities; dark blue reflecting high signal intensity, white-grey reflecting weak signal intensity. Light blue corresponds to genomic regions containing highly repetitive sequence. (C) Aggregate plot of signals corresponding to the regions indicated by the brackets in B.

Of 10483 CHD7 binding sites identified at the medium confidence threshold, 16.4% (1723) are located within 1.5 kb of a transcriptional start site (TSS). Of the remaining CHD7 sites, 46.0% (4819) are intergenic, 2.3% (239) are located within exons, and 35.3% (3702) are intronic (Figure 1C). The distribution of CHD7 is similar at the lower and higher thresholds, although fewer CHD7 sites are found at TSSs at high threshold (7.8% versus 16.4%). This discrepancy is due to differences in CHD7 signal intensity, i.e., CHD7 signals at TSSs are generally lower than at distal regions (Figure 2B and 2C), causing signals at TSSs to “drop out” when thresholds are increased. The rest of the analyses were performed using the 10483 CHD7 sites identified at the medium threshold.

### CHD7 binding sites have similar characteristics to gene enhancer elements

Based on previous studies suggesting that CHD7 binds enhancers [9], we implemented ChIP-seq on mouse ES cells to map the genome-wide distribution of P300 (NM_177821), a known enhancer-binding protein [19]. In addition, we generated a genome-wide map of open chromatin in mouse ES cells using the technique of DNase-seq [20]. The location of the P300 sites and the open regions of chromatin were compared to the distribution of CHD7, along with the locations of the following seven different histone modifications previously mapped by ChIP-seq: H3K4me1, H3K4me2, H3K4me3, H3K9me3, H3K27me3, H3K36me3, and H4K20me3 [21], [22]. The strategy for comparing these datasets is outlined in Figure 2A and the results are plotted as a heatmap in Figure 2B. The heatmap reveals several distinct clusters defined by the presence or absence of specific histone marks and/or factors. Sites containing the most robust CHD7 signals cluster in the upper third of the heatmap. These sites show features of enhancer elements, including high levels of H3K4me1, H3K4me2, and P300. The CHD7 sites are also contained within open regions of chromatin that are hypersensitive to DNase I digestion (DNase HS). In comparison, the cluster in the lower portion of the heatmap, which has lower levels of CHD7, display the characteristic features of promoters. Specifically, these sites have high levels of H3K4me3 and H3K4me2, and are DNase HS. As previously described, these promoter regions also contain low levels of H3K4me1 as a distinctive bimodal peak centered over the TSS [23]. Also as expected for promoters in this cluster, the level of H3K36me3 is low at TSSs and high in the bodies of genes undergoing transcriptional elongation [24]. CHD7 is absent from clusters containing histone marks generally associated with gene repression, including H3K9me3, H3K27me3, and H4K20me3 [25]. Overall, these results are consistent with CHD7 binding to a subset of enhancer elements and, to a lesser extent, promoter regions. This is also apparent when the regions identified as enhancers and promoters are aggregated and plotted (Figure 2C). The difference between CHD7 signals at enhancers and promoters could reflect recruitment of CHD7 to enhancers and subsequent transient association with promoters via looping. Further studies are required to test this looping model.

### CHD7 co-localizes with P300, OCT4, SOX2, and NANOG at active gene enhancer elements

The locations of the following 13 transcription factors were recently mapped by ChIP-Seq: NANOG (NM_028016), OCT4 (also called POU5F1, NM_013633), STAT3 (NM_213659), SMAD1 (NM_008539), SOX2 (NM_011443), ZFX (NM_011768), c-MYC (NM_010849), n-MYC (NM_008709), KLF4 (NM_010637), ESRRB (NM_011934), TCFCP2L1 (NM_023755), E2F1 (NM_007891), and CTCF (NM_181322) [26]. Interestingly, iterative pairwise comparisons between all 13 datasets indicated that specific sites in the genome are extensively co-occupied by multiple transcription factors. Genomic segments bound by 4 or more factors were termed multiple transcription factor loci, or MTLs. MTLs are further distinguishable by distinct combinations of proteins. For example, NANOG, OCT4, SOX2, SMAD1, and STAT3 tend to co-localize to one set of MTLs, while c-MYC, n-MYC, ZFX, and E2F1 co-occupy different MTLs. Interestingly, 25 out of 25 loci co-occupied by NANOG, OCT4, SOX2, SMAD1, and STAT3 functioned as enhancers when placed downstream of a luciferase reporter. By comparison, 0/8 constructs containing genomic fragments co-bound by proteins in the Myc cluster activated the luciferase reporter. These data, along with correlations to corresponding gene expression data, indicate that ES cell-specific gene expression is mediated by combinatorial binding of OCT4, SOX2, NANOG, SMAD1, and STAT3 to enhancer elements.

**Figure 3:**
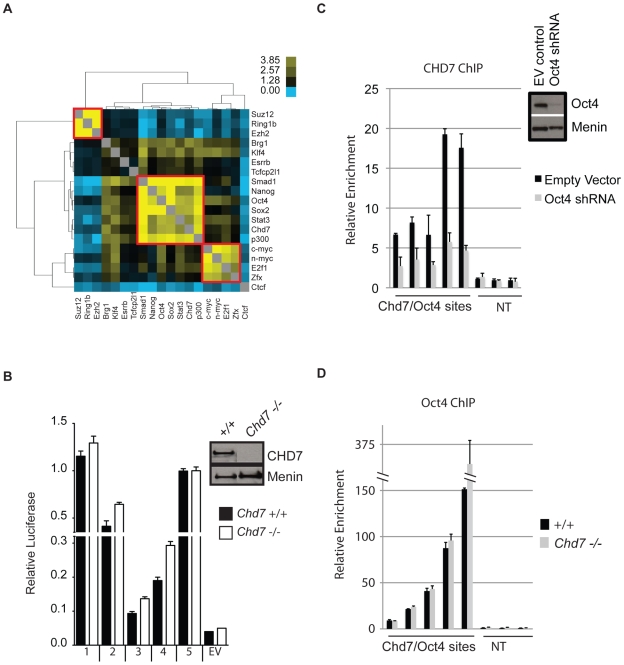
CHD7 co-localizes with core ES cell factors at functional enhancer elements. (A) Co-localization of transcription factors. Colors in the heat map correspond to the colocalization frequency of each pair of factors; yellow reflecting high correlation, and blue reflecting little or no correlation. The three clusters referred to in the text are highlighted in the red boxes. (B) Genomic fragments from CHD7/OCT4 MTLs show CHD7-independent enhancer activity. Sites from the CHD7/OCT4 MTLs were cloned into a luciferase reporter vector and assayed for enhancer activity in both in wild type (+/+) and *Chd7* null (−/−) ES cells. The western blot (upper right) shows the levels of CHD7 protein in wild type and *Chd7* null ES cells. MENIN is a nuclear protein that serves as a loading control [49]. No enhancer activity was observed upon transfection of constructs into mouse embryonic fibroblasts (not shown). EV  =  empty vector control. (C) CHD7 occupancy is dependent on OCT4. CHD7 ChIP at five CHD7/OCT4-MTLs is shown in ES cells transfected with either *Oct4* shRNA or a control RNAi construct (EV control). NT  =  non-target control region. The levels of OCT4 in control and *Oct4*-shRNA transfected cells are shown in the western blot (upper right). (D) OCT4 occupancy is not dependent on CHD7. OCT4 ChIP at the same five MTLs as in panel C is shown in wild type and CHD7 null cells.

We tested whether CHD7 co-localizes to enhancer elements with any of the previously mapped factors in ES cells. To do this, pairwise comparisons were made between the binding sites of CHD7, P300, the 13 factors listed above, and the following four factors for which public data is available: SUZ12 (NM_199196), RING1B (NM_011277), EZH2 (NM_007971), and BRG1 (NM_011417) [27], [28]. Odds ratios representing the correlation between binding sites for each pair of factors were calculated, hierarchically clustered, and plotted in heatmap (Figure 3A). Using this strategy, we identified 3 clusters of proteins that co-localize to specific loci within the ES cell genome. The smallest cluster is defined by proteins that comprise the Polycomb-repressive complexes, and includes SUZ12, RING1B and EZH2. The next smallest cluster is identical to that mentioned above, and contains c-MYC, n-MYC, E2F1, and ZFX. The largest cluster is defined by the presence of both CHD7 and P300 and the five factors previously shown to colocalize to functional enhancers: OCT4, SOX2, NANOG, SMAD1, and STAT3. In contrast, the insulator binding protein CTCF did not show strong association with any of the factors [29]. We used ChIP-PCR assays to validate the presence of CHD7 at six loci that showed co-occupancy of MTLs containing OCT4. As a control for specificity, CHD7 binding at these sites was assayed in ES cells harboring a homozygous nonsense mutation in the *Chd7* gene (W973X) and shown to be negative (Figure S1).

Five CHD7-OCT4 MTLs were tested for enhancer activity using a luciferase reporter assay. All five constructs showed robust activity in ES cells and this activity was ES cell specific (Figure 3B). These constructs were also capable of activating luciferase in CHD7 null mouse ES cells, suggesting that CHD7 is dispensable for enhancer activity at these MTLs. Collectively, these findings indicate that CHD7 is associated with core components of the transcriptional circuitry of ES cells that functions to mediate ES cell-specific gene expression via an enhancer-binding mechanism. Although CHD7 appears to be dispensable for enhancer activity, we cannot rule out the possibility that CHD7 sites outside of the five tested are dependent on CHD7, or if genes that are regulated by multiple, cooperating enhancers are influenced by loss of CHD7. We also cannot exclude the possibility that enhancer-activity is dependent on CHD7 at later stages in development, in cell types that are more relevant than ES cells to the phenotype of CHARGE syndrome.

We next tested whether binding of CHD7 is dependent on OCT4 binding to MTLs. CHD7 ChIP was performed at five CHD7/OCT4-MTLs in ES cells transfected with *Oct4* shRNA. The data show that CHD7 binding is diminished upon knockdown of OCT4 (Figure 3C), indicating that OCT4 is required for the binding of CHD7. To test if OCT4 binding is dependent on CHD7, OCT4 ChIP was performed at the same five MTLs in wildtype and *Chd7* null ES cells. The results show that CHD7 is not required for binding of OCT4 (Figure 3D). These results not only validate combinatorial binding of these specific factors, but also are consistent with published data indicating that OCT4 is the key factor required for stabilizing complex formation at functional enhancer elements [26].

### CHD7 co-immunoprecipitates with P300

**Figure 4:**
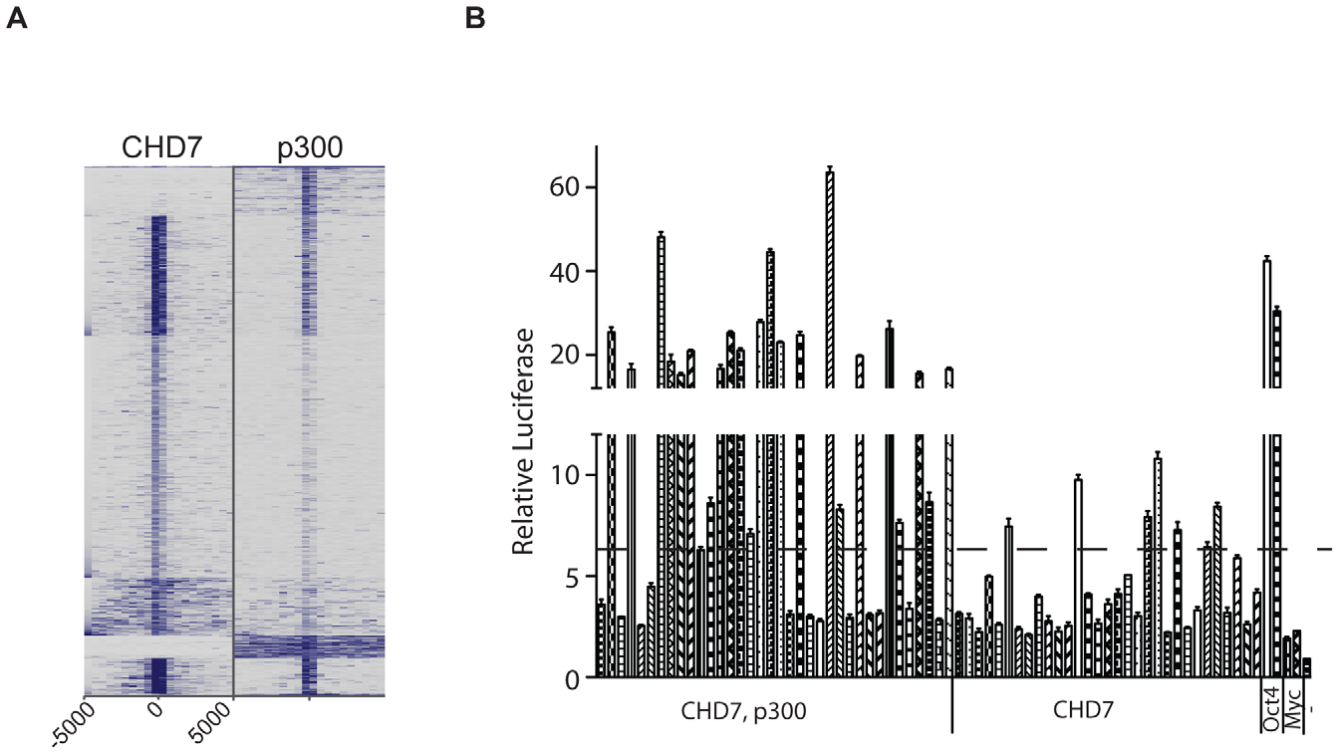
CHD7 sites bound and not bound by P300 can activate transcription. (A) Comparison of 1000 randomly selected P300 sites to 1000 randomly selected CHD7 sites. (B) Enhancer assay. OCT4 sites correspond to MTLs that were previously shown to activate the luciferase reporter gene and serve as positive controls. MYC sites correspond to Myc MTLs that fail to activate the luciferase reporter gene and serve as negative controls. -, untransfected.

Given the strong evidence that P300 binds to functional enhancers [19], we further delineated the relationship between CHD7 and P300 binding by comparing 1000 randomly selected CHD7 and P300 binding sites using the approach outlined in Figure 2A. A heatmap can reveal signals that are below threshold but above background, and therefore this plot is far more informative than a Venn diagram. The heatmap indicates that the most robust CHD7 binding sites are also shared by P300, although a number of robust P300 sites altogether lack CHD7. Weaker, but significant CHD7 binding sites harbor little to no P300 binding (Figure 4A). A direct interaction between CHD7 and P300 was then tested by co-immunoprecipitation. P300 was successfully co-immunoprecipitated with antibodies to CHD7, although reciprocal co-IP of CHD7 with P300 antibodies was not observed (Figure S1).

### Delineation of enhancer activity at CHD7 sites bound and not bound by P300

We selected 67 CHD7 binding sites and cloned them downstream of a luciferase reporter driven by the *Oct4* minimal promoter. 36 of the 67 sites were cobound by P300 and CHD7, and the remaining 31 showed minimal or undetectable P300 binding. Upon transfection into wildtype ES cells, 23/36 (63.8%) of the sites bound by both P300 and CHD7 showed greater than a 3-fold increase in luciferase activity over negative controls (Figure 4B). Using this same threshold, 7/31 (22.6%) sites bound by CHD7 alone showed enhancer activity, and the level of activity was less robust than that determined for sites bound by both P300 and CHD7. Overall the data suggest that highly active enhancers contain P300 and are consistent with previous studies indicating that P300 accurately predicts enhancer function. The data also indicate that sites bound by CHD7 and not P300 represent a subset of functional enhancers that generally show modest activity.

### ES cell-specific gene expression correlates with CHD7 occupancy at gene enhancers

**Figure 5:**
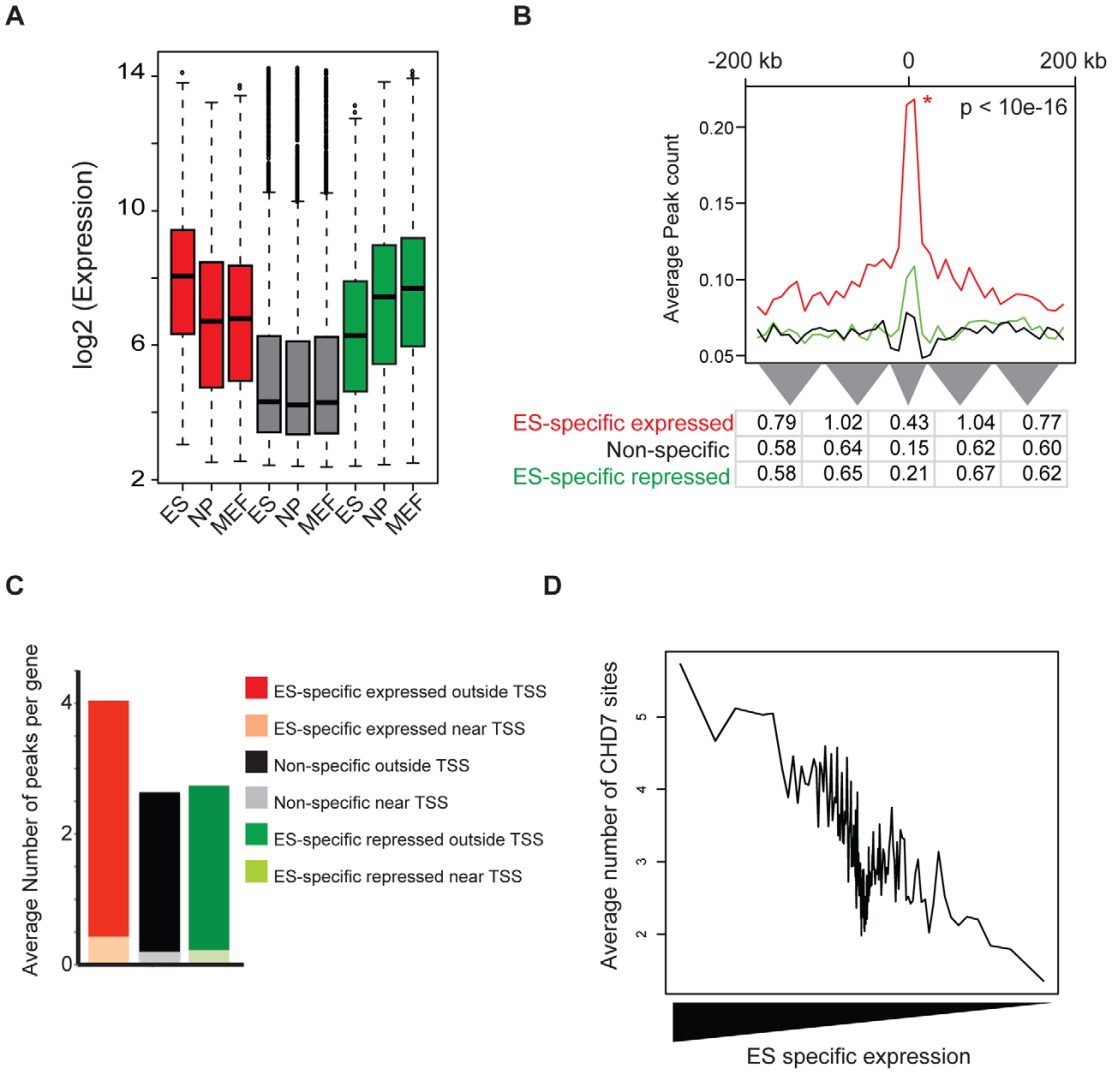
CHD7 binding correlates with ES cell specific expression patterns. (A) Distribution of expression of genes within each set. Mouse genes were classified as ES-specific expressed (red), ES-specific repressed (green), and non-specific (gray) based on the specificity of their expression in ES cells, as compared to neural precursor (NP) and mouse embryonic fibroblasts (MEF). (B) CHD7 localization relative to ES-specific expressed genes (red), non-specific expressed (black), and ES-specific repressed (green). The P-value reflects significance of CHD7 enrichment of ES-specific expressed genes compared to non-specifically expressed genes, as determined by a Wilcoxon signed-rank test. Average peak count refers to the average number of CHD7 binding sites at various distances within 200-kb of the TSS of the genes in each set. The table below the plot shows the average number of binding sites per gene within smaller intervals defined by the width of the grey triangles. (C) Average number of CHD7 binding sites per gene in each gene set. CHD7 sites within 20 kb of a TSS are distinguished from sites located between 20 and 200 kb. (D) Comparison of CHD7 enrichment and ES cell-specific gene expression.

Given that enhancers function to mediate tissue-specific gene expression, we hypothesized that CHD7 sites identified by ChIP-seq would positively correlate with ES cell-specific gene expression. To test this hypothesis, we utilized genome-wide expression data from multiple cell types to group genes that are: (1) specifically expressed in ES cells, (2) specifically repressed in ES cells, and (3) non-specific to ES cells. These gene sets were generated by comparing global gene expression levels between mouse ES cells, neural precursors (NP) derived from ES cells, and embryonic fibroblasts (MEF) and computing a tissue-specificity score for each gene using Shannon-entropy [30]. The distribution of expression of genes within each set is shown in Figure 5A. We then calculated and plotted the average number of CHD7 binding sites within 200 kb of the TSS of each gene in each set (Figure 5B and 5C). The results indicate that genes with ES cell-specific expression have far more CHD7 binding sites than genes that are specifically repressed in ES cells or not specific to ES cells. Similar results are obtained when genes are ranked by the specificity of their expression in ES cells and plotted against the number of CHD7 binding sites (Figure 5D). Collectively, the results show a very strong correlation between CHD7 binding and ES-specific gene expression.

### ES cell self-renewal, pluripotency, and somatic reprogramming are not dependent on CHD7 function

The data above indicate that CHD7 co-localizes with components of the core transcriptional circuitry in ES cells, including OCT4, SOX2, and NANOG. Moreover, CHD7 binds to loci encoding proteins proposed to mediate ES cell self-renewal and pluripotency, including OCT4, SOX2, and NANOG, as well as DPPA2 (NM_028615), DPPA4 (NM_028610), MYC, and SALL4 (NM_201396) (Figure S2) [31]. However, *Chd7* null mice die in mid-gestation, far beyond the ES cell stage. We therefore would not necessarily expect the loss of CHD7 to overtly affect the functions of ES cells. Nevertheless, we tested whether the processes of ES cell self-renewal, pluripotency and/or somatic reprogramming are affected by the loss of CHD7.

*Chd7* null ES cells do not spontaneously differentiate in culture or exhibit any detectible growth defects (data not shown). Moreover, the levels of *Oct4*, *Sox2*, and *Nanog* are similar between wildtype and *Chd7* null ES cells (Figure S3). These results indicate that the processes of ES cell self-renewal are not overtly affected by absence of CHD7. To test if absence of CHD7 affects pluripotency, wildtype and *Chd7* null ES cells were differentiated into embryoid bodies (EBs) and multiple gene markers for endoderm, ectoderm, and mesoderm formation were quantified by qRT-PCR (see Materials and Methods). In addition, the levels of *Oct4*, *Sox2*, and *Nanog* were quantified to determine if CHD7 influences the rate of differentiation. *Chd7* levels increase at day 4 of EB formation and remain high throughout EB formation (Figure S3). All other genes tested responded as expected over the course of EB formation, and no significant expression differences were observed between wildtype and *Chd7* null cells (Figure S3 and data not shown). Lastly, we tested if absence of CHD7 affects somatic cell reprogramming by generating inter-species heterokaryons with *Chd7* null mouse ES cells [32]. Similar to wildtype ES cells, *Chd7* null ES cells fused to human B cells activated pluripotency genes including h*Oct4* (NM_002701), h*Nanog* (NM_024865), h*Cripto* (NM_003212), and h*Dnmt3b* (NM_006892) (Figure S5). Collectively, the results indicate that CHD7, despite being associated with the core transcriptional circuitry in ES cells, is not essential for the processes of ES cell self renewal, pluripotency, or somatic cell reprogramming.

### CHD7 functions as a repressive modulator of ES cell–specific gene expression

**Figure 6:**
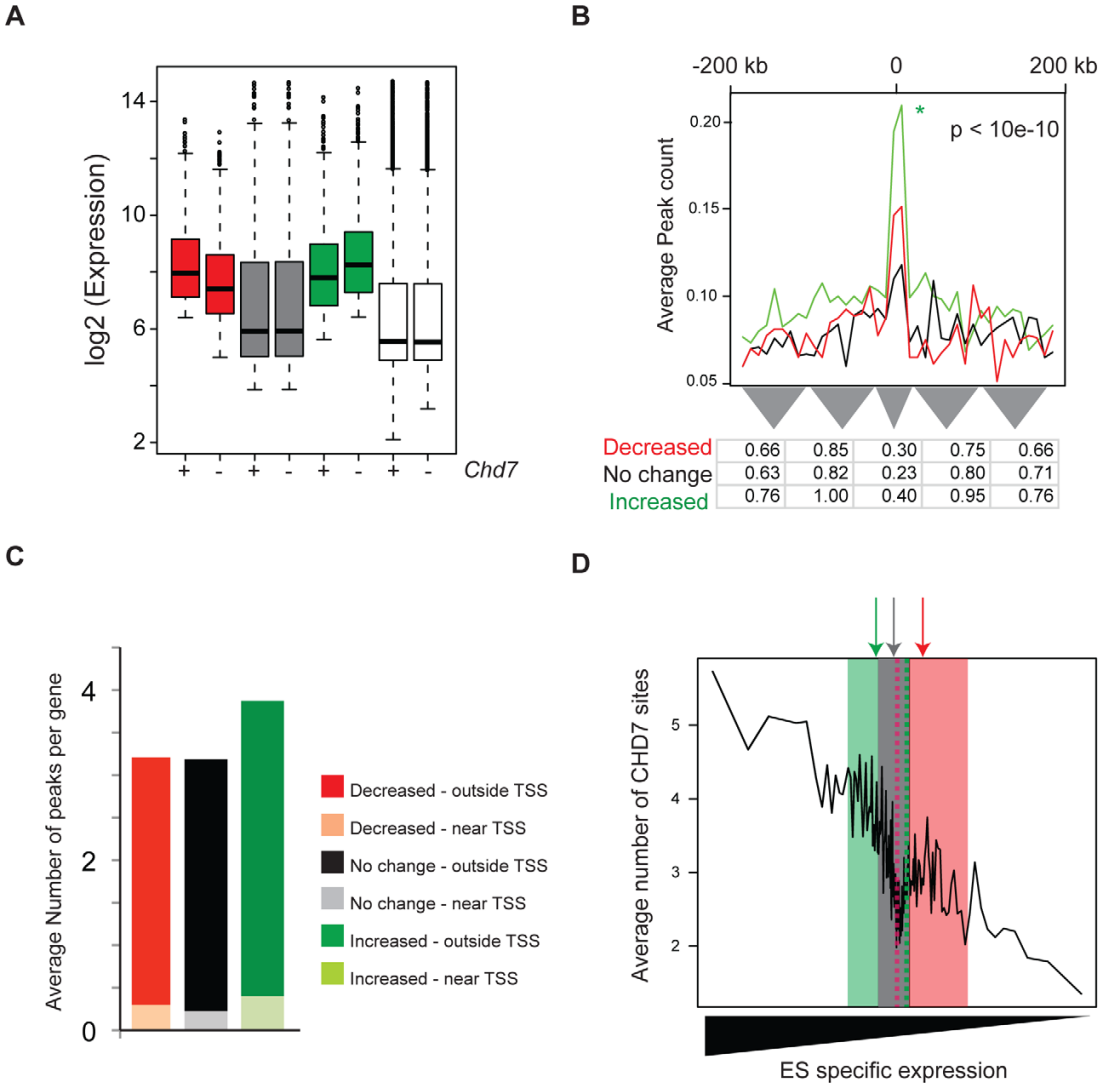
CHD7 modulates ES cell specific expression. (A) Distribution of gene expression in wild type (+) and CHD7 null (−) ES cells. Genes were defined as decreasing (red, n = 800), increasing (green, n = 1200), or not changing (gray, n = 1000) upon loss of CHD7. The white boxplots represent the genome-wide distribution of gene expression in either wild type or *Chd7* null ES cells. (B) CHD7 is significantly enriched near genes that increase in expression upon loss of CHD7. The P-value reflects significance of CHD7 enrichment of differentially increased genes (green) compared to non-differentially expressed genes (black). Comparisons between differentially decreased genes (red) and non-differentially expressed genes were insignificant. Similar results are obtained when CHD7 sites are correlated to smaller gene sets containing the top 100, 200, or 400 differentially expressed genes (data available upon request). (C) Average number of CHD7 binding sites per gene for each gene set. CHD7 sites located near TSSs are distinguished from those located distal to TSSs. (D) To determine whether differentially expressed genes are ES cell specific, genes in each of the 3 classes above were scored according to the specificity of their expression in ES cells, as compared to NP and MEFs. The distribution of the ES-specificity scores for genes in each of the 3 sets was then superimposed on the plot from Figure 4D. Note that ES cell specificity scores for genes that increase upon loss of CHD7 (green) are located on the left side of the plot, compared to genes that either decrease (red) or are not differentially expressed (gray). Arrows correspond to median specificity scores for each gene set.

To determine the role of CHD7 at enhancers, we obtained global gene expression profiles from *Chd7* wildtype, heterozygous, and null ES cells. Using methodology similar to that used to generate ES-specific gene sets, we classified genes as decreased, increased or not differentially expressed between wildtype and *Chd7* null ES cells (Figure 6A). Interestingly, genes that are differentially expressed reside within the upper range of the overall distribution of ES cell gene expression, indicating that genes influenced by loss of CHD7 are generally expressed at relatively high levels (Figure 6A, compare red and green boxes to the white boxes). Similar to above, we then counted and plotted the average number of CHD7 sites within 200 kb of the TSS of each gene within each set. The results indicate that significantly more CHD7 sites are located near genes that increase upon loss of CHD7 than genes that decrease or are not differentially expressed (Figure 6B and 6C; compare green plot to red and black plots). Moreover, genes that increase upon loss of CHD7 are more ES cell specific than the genes in the other two categories (Figure 6D). The significant correlation between CHD7 occupancy and reduced gene expression suggests that CHD7 functions to limit the expression of a subset of ES-specific genes. Moreover, because the loss of *Chd7* results in relatively modest expression changes of genes that are already highly expressed, the repressive action of CHD7 is modulatory in nature. Similar results are observed in comparisons between wildtype and *Chd7* heterozygous cells, as well as comparisons between heterozygous and null ES cells (Figure S4). Thus, the association between CHD7 binding and repressive modulation of ES cell-specific expression is unlikely to be due to ES clone-specific effects, and additionally indicates that CHD7-mediated regulation is dosage dependent.

### CHD7 sites near differentially repressed genes show similar characteristics to those located elsewhere in the genome

**Figure 7:**
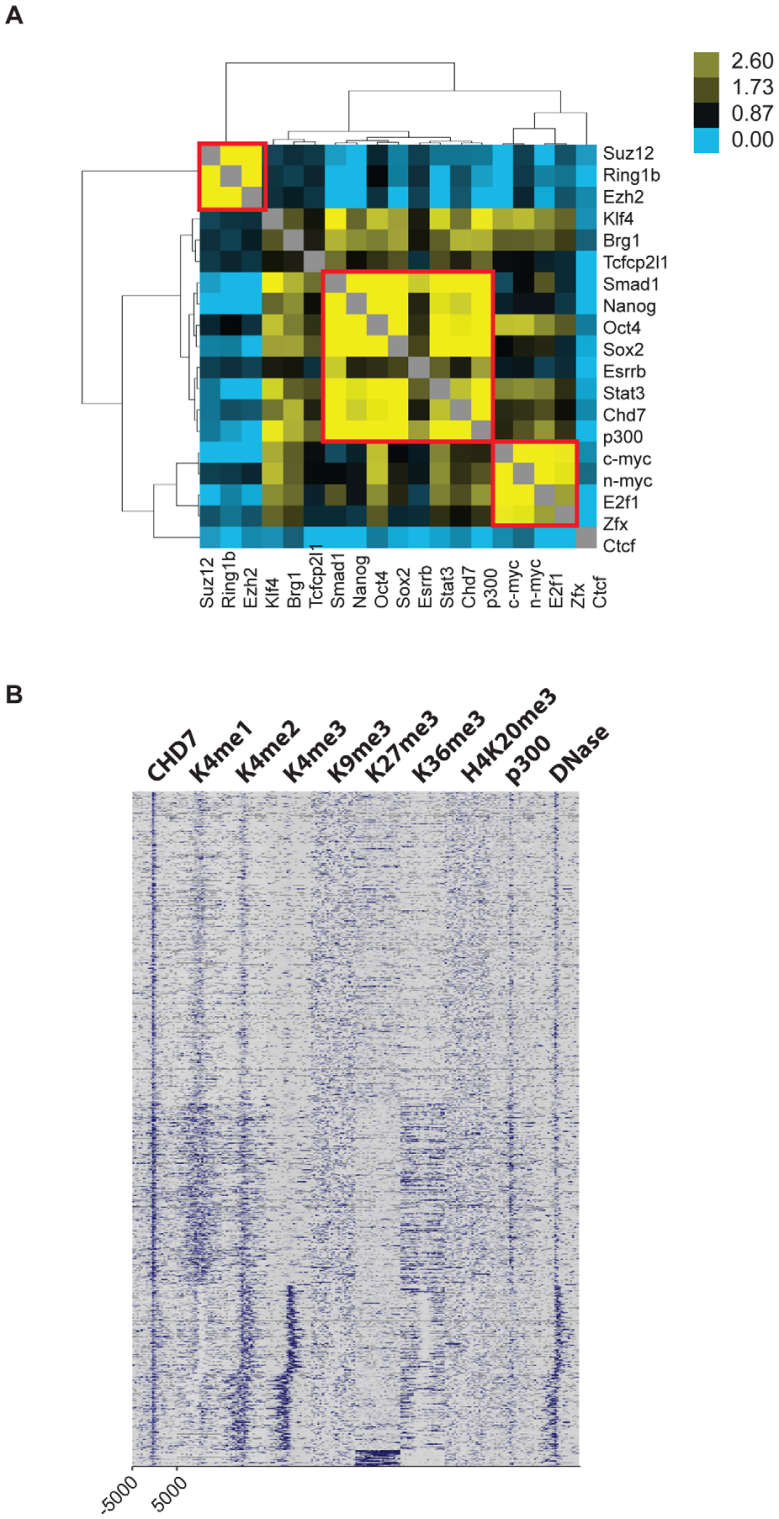
CHD7 binding sites near CHD7-regulated genes show the features of enhancer elements. (A) Colocalization of proteins near genes negatively regulated by CHD7. Similarly to CHD7 sites located elsewhere in the genome, CHD7 colocalizes with P300, OCT4, SOX2, NANOG, SMAD1 and STAT3 at regions associated with CHD7-mediated regulation. (B) CHD7 sites associated with negative modulation (n = 3118) were correlated to histone modifications, P300 binding sites, and open chromatin, as in Figure 2.

We selected CHD7 sites located within 200 kb of the differentially repressed genes in wildtype ES cells and examined them in detail. Similar to sites located across the entire genome, CHD7 sites at repressed genes are frequently co-occupied by P300, OCT4, SOX2, NANOG, SMAD1, and STAT3 (Figure 7A). Furthermore, although we do detect a slightly higher proportion of CHD7 sites at promoters containing H3K4me3 (∼25% versus ∼16%), sites near the genes subject to CHD7-directed negative modulation have similar characteristics to those found elsewhere, i.e., most contain high levels of P300 and H3K4me1/2, relatively low levels of H3K4me3, and are contained within open chromatin that is hypersensitive to DNase I digestion (Figure 7B). These findings support the notion that the repressive modulatory action of CHD7 is indeed related to its binding to enhancer elements, rather than a different type of functional element.

## Discussion

**Figure 8:**
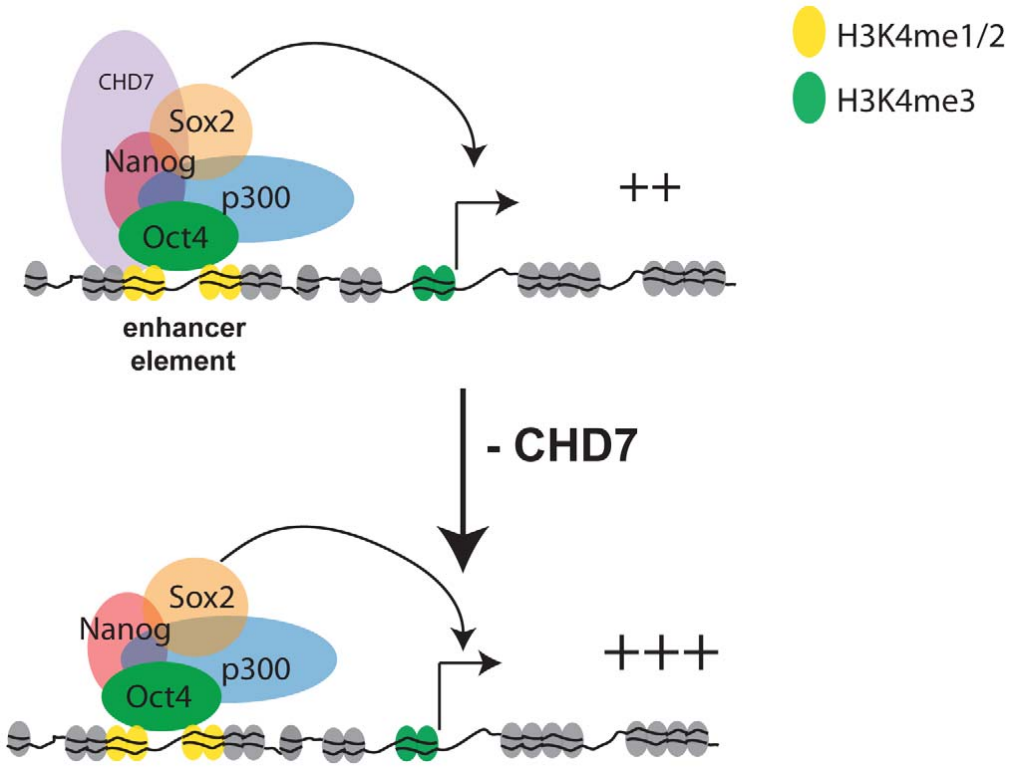
Model for CHD7-mediated transcriptional modulation in ES cells. CHD7, via its tandem chromodomains, binds to functional gene enhancers marked with mono- and di-methylated K4 of histone H3. In ES cells a subset of the CHD7 sites are cobound by P300, OCT4, SOX2, and NANOG. Collectively, these proteins coactivate gene expression through enhancer-promoter interactions. A subset of CHD7 sites that are not bound by P300, OCT4, SOX2, and NANOG can also enhance transcription, although the identity of the CHD7-associated factors at these sites is not known (not shown). Reduction of CHD7 levels results in increased transcription of a subset of ES cell-specific genes that are already expressed at reasonably high levels, suggesting an antagonistic role for CHD7 at enhancers. We hypothesize that CHD7 functions similarly at later stages in development, which would imply that dysregulated expression of tissue-specific genes contributes to the pathogenesis of CHARGE syndrome.

The adoption of genome-wide approaches for mapping transcription factors and histone-modifications by the ENCODE consortium and other groups has helped to rapidly identify the genomic locations of functional elements and their characteristics. As demonstrated here for CHD7, these efforts are facilitating functional characterization of chromatin-associated proteins, because once generated, multiple datasets can be compared to infer a protein's function. However, as more and more factors are mapped, it is becoming increasingly apparent that multiple proteins often co-occupy a given functional element, and the functional significance of this is unclear. For instance, our studies and others indicate that at least 12 factors bind to the distal enhancer of *Oct4* in ES cells [26]. Some factors are clearly essential for maintaining normal cell function. For example, reduction of OCT4 [33], SOX2 [34], or SALL4 [35] results in rapid differentiation of ES cells, indicating that these proteins play critical roles in the ES cell circuitry to maintain self-renewal and pluripotency. Our studies indicate that CHD7, although not a critical component of the ES cell circuitry, functions at enhancers to modulate the expression levels of ES-specific genes. The modulation can occur in either the positive or negative direction, however negative-regulation appears to be the more direct effect of CHD7 binding. This modulatory role suggests that regulation of tissue-specific gene expression involves the coordinated combinatorial binding of not only potent regulators that switch genes on and off, but also factors that mediate fine-tuning. A model for CHD7 function is shown in Figure 8.

The mechanism by which CHD7 modulates transcription in ES cells is currently unknown and will be the subject of future investigation. However, it is well established that chromatin-remodeling proteins exist in large multi-subunit complexes, and the composition of proteins within these complexes determines how these proteins control transcriptional programs and establish cellular identity [36]–[38]. As this manuscript was under review, CHD7 was found to physically associate with PBAF (polybromo- and BRG1-associated factor containing complex) [39]. Through our colocalization analyses, we detected overlap between sites occupied by CHD7 and BRG1 (Figure 3A), although the extent of overlap was not as significant as that for other factors. Interestingly, BRG1 was shown through ChIP-seq studies to colocalize to chromatin with OCT4, SOX2, and NANOG in ES cells, and to both positively and negatively regulate transcription within this circuitry [27]. Therefore, one possibility is that the mechanism of CHD7 as both a positive and negative regulator is related to its interactions with BRG1-containing complexes. However, we also found that a substantial fraction of CHD7 sites do not contain BRG1, and we therefore cannot rule out the possibility that CHD7 cooperates with other, currently unidentified proteins to regulate transcription. CHD7 co-localization analyses with factors in addition to the 18 we tested, as their binding profiles become available, could help reveal these interactions.

How might haploinsufficiency of CHD7 give rise to CHARGE syndrome? We hypothesize that dysregulated tissue-specific gene expression is the underlying cause. This hypothesis is supported not only by the evidence presented here, suggesting a role for CHD7 as a modulator of transcription in ES cells, but also by previous studies suggesting that CHD7 binds to enhancer elements in differentiated cell types [9]. As in ES cells, the effect of reduced CHD7 levels on transcription may be modest during development. Modest effects could translate into dramatic effects that perturb development, particularly if CHD7 directly regulates a critical transcription factor. However, the possibility that haploinsufficiency of CHD7 induces large transcriptional effects at time points beyond the ES cell stage still needs to be tested. Given that the affected tissues in CHARGE syndrome are derived from multiple germ layers, we also cannot rule out the possibility that the subtle expression changes occurring at the ES cell stage could themselves contribute to the phenotype, although this scenario is unlikely given that *Chd7*-null ES cells are capable of differentiating into all three germ layers. Future studies aimed at investigating gene expression patterns in relevant tissues from CHD7 mutant mice could help shed light on these and other possibilities. In that regard, defects in neural crest cell migration were recently proposed to underlie the anomalies in CHARGE syndrome [39]. Thus, neural crest cells might serve as excellent resource for identification of critical CHD7 target genes. The data presented here suggest that the critical target genes are likely to be neural crest-specific, and may be either upregulated or downregulated inappropriately when CHD7 is haploinsufficient.

## Materials and Methods

### Ethics statement

Studies involving mice are approved by the CWRU Animal Care and Use Committee.

### ChIP–Seq and DNase–Seq

R1 ES cells were cultured under feeder-free conditions as previously described [9]. Chromatin preparation, ChIP, DNA purification, and library preparation for Illumina sequencing were performed as described [40]. ChIP was performed using commercially available antibodies to CHD7 (Abcam, ab31824) and P300 (Santa Cruz, sc-585). Sequencing was done on an Illumina GAII instrument according to the manufacturer's protocol. For CHD7 and P300, 9,154,400 and 19,480,925 unique reads were obtained, respectively. The Eland software (Illumina) was used to align reads with up to two mismatches against the mm8 reference genome. Regions significantly enriched for CHD7 or P300 binding were identified using F-seq, a feature density estimator for high-throughput sequence tags [41]. Genomic regions found to have an unexpectedly high percentage of reads aligning to the sample positions, indicating PCR artifacts, were eliminated from the analysis. Also excluded from the analysis were CHD7 peaks in which the midpoint overlapped a repetitive region. DNase-Seq was performed as previously described [42]. Sequences obtained from 8 lanes of sequencing on an Illumina GAII instrument (38,342,306 reads) were aligned to the mouse genome (mm8) using MAQ [43], and peaks corresponding to DNase HS sites were determined using F-seq. All data, including the list of CHD7, P300, and DNase HS peaks will be deposited in GEO upon publication.

### Cluster analysis

For cluster analyses in Figure 2, 100 high-confidence peaks on mouse chromosome 19 were randomly selected from DNase-seq data, CHD7 and P300 ChIP-seq data, and the following seven publically available ChIP-seq datasets: H3K4me3, H3K9me3, H3K27me3, H3K36me3, H4K20me3 (GSE12241); H3K4me1, H3K4me2 (GSE11172) [21], . A 10 kb window centered on the midpoint of each peak was then generated. The 10 kb region was divided into 20 bins of 500 bp, and an enrichment value corresponding to the median number of sequence reads in each bin was calculated. To allow for comparisons between factors with different normal distributions, data were standardized using a Z-score transformation. Normalized data from each ChIP-seq/DNase-seq dataset were then aligned in parallel columns to create a 1000 row×10 column matrix. The data in the rows were then K-means clustered (Euclidian distance, 1000 runs, 5 clusters) in Gene Cluster 3.0 [44]. Clusters were visualized with Java Treeview [45].

### Generation of wild-type and *Chd7* mutant ES cell lines

Timed matings between male and female heterozygous *Whirligig* mice [8], strain C3HeB/FeJ, were set up. Whirligig mice harbor a G2918A transition in exon 11 of the *Chd7* gene, resulting in a W973X nonsense mutation. From the inner cell mass of fourteen blastocysts harvested from pregnant females, one wild type, three *Chd7* heterozygous and two *Chd7* homozygous lines ES cells were generated as previously described [46].

### Differentiation of ES cells to embryoid bodies

One *Chd7* wildtype and two *Chd7* ^−/−^ ES cell lines were differentiated into embryoid bodies according to standard protocols. Cells were harvested at days 0, 2, 4, 7, and 10 during EB formation. The following genes were assayed in triplicate by standard qRT-PCR using SyBr green detection: *Chd7*, *Gapdh*, *beta-actin*, *Oct4*, *Sox2*, and *Nanog*. The following germ layer-expressed genes were assayed: *Sox1* and *Fgf5* (ectoderm); *Gsc* and *T* (mesoderm); *Afp*, *Sox17*, and *Gata6* (endoderm); *Sox7* and *Hhex* (visceral endoderm). Primer sequences are available upon request.

### Microarray analyses

Expression datasets for mouse ES, neural precursor (NP), and embryonic fibroblast cells were downloaded from Gene Expression Omnibus (Accession number: GSE8024) [22]. Raw data were RMA normalized using the R Affy package [47] available in Bioconductor [48]. *Chd7* wildtype, heterozygous, and homozygous ES cells derived from preimplantation embryos were grown on feeder cells and total RNA was isolated using Trizol. The ratio of ES to feeder cells was estimated at 5∶1. RNA was labeled and hybridized to Illumina Mouse Ref-8 v2 Expression BeadChIP microarrays according to the manufacturer's instructions. Raw data were background subtracted and quantile normalized using Illumina Bead Studio software.

### Generation of gene sets

Shannon entropy was used to rank genes by the specificity of their expression in ES cells compared to NP cells and MEFs [30]. To generate a list of ES-specific expressed genes, genes were first sorted by their categorical tissue-specific values from most to least ES cell specific. The entire gene list was then divided into blocks of 400 genes. Expression levels of the top 400 genes were then compared between ES, NP and MEFs using a T-test. This process was repeated for subsequent gene blocks until a significant difference in expression (P<0.05) was no longer achieved. This occurred between blocks 11 and 12, encompassing 4500 genes that we consolidated into one list of ES-specific expressed genes. The list of ES-specific repressed genes was generated in the same manner, except that genes were first sorted by their Shannon entropy scores from least to most ES cell-specific. This process yielded 4469 ES-specific repressed genes. 4000 genes in the middle of the list and not significantly differentially expressed among ES cells, NP cells and MEFs were selected for the set of non-differentially expressed genes. A similar approach was used to define genes that were decreased, increased, and not differentially expressed between *Chd7* wildtype, heterozygous, and null ES cells. A total of 800 genes in wildtype ES cells were significantly decreased in expression in *Chd7* null ES cells (p<0.00003). 1200 genes in wildtype ES cells were significantly increased in expression in *Chd7* null ES cells (p<0.003). 1000 genes that were not significantly different between wildtype and null cells were used for the list of non-differentially expressed genes (p>0.05). The identity of the genes within each set, as well as their respective fold changes and corresponding number of CHD7 sites are listed in Table S1, S2, and S3.

### Generation of colocalization maps

The following ChIP-seq datasets were downloaded from GEO: OCT4, SOX2, NANOG, SMAD1, KLF4, ESRRB, CTCF, n-MYC, c-MYC, STAT3, E2F1, TCFCP2L1, ZFX (GSE11431) [26]; BRG1 (GSE14344) [27]; SUZ12, EZH2, RING1B (GSE13084) [28]. The binding sites for these factors in addition to CHD7 and P300 (19 in total) were assembled into one list. Sites located within 200 bp of each other were consolidated. The final list contained 121,362 unique binding sites. Each binding site was then examined for the presence of each of the 19 factors. An odds ratio from a Fishers Exact test, representing the correlation between binding sites for each pair of factors, was then calculated. Odd ratios were organized in a 19×19 matrix and hierarchically clustered using Cluster 3.0 and data were visualized in Java TreeView.

### Luciferase assays

Constructs containing CHD7/OCT4 MTLs located downstream of a *Pou5f1* minimal promoter driving luciferase were kindly provided from Huck-Hui Ng (Genome Institute of Singapore). The coordinates for the CHD7/OCT4 MTLs are as follows (mm8): chr7:11914503-11914844, chr5:103964736-103965061, chr8:75263314-75263637, chr8:50250752-50251069, chr16:84651775-84652095. The coordinates corresponding to the 67 CHD7 sites tested in Figure 4 are listed in Table S4 in the same order as shown in Figure 4. Constructs containing MTLs were transfected in triplicate into *Chd7* wildtype and null ES cells using Lipofectamine 2000 (Invitrogen). Constructs containing the 67 CHD7 sites were transfected into R1 ES cells. In all instances a renilla luciferase plasmid (pRL-SV40 from Promega) was cotransfected as an internal control. Media was replaced after 24hrs with fresh media and cells were harvested after a total of 48hrs. Reported luciferase expression levels are relative to internal renilla control.

### OCT4 RNAi

Wild type ES cells were transfected with an *Oct4* shRNA construct commercially available from (Oligoengine) as previously described (Chen et al 2008). Puromycin selection was introduced 1 day after transfection, and the cells were crosslinked and harvested for ChIP 48 hours after transfection. pSuper-puro empty vector was used as negative control. OCT4 knockdown did not affect the level of CHD7 protein, as determined by western blot.

### ChIP–PCR

ChIP reactions were performed as previously described [9]. OCT4 ChIP was performed using sc-8628 antibody from Santa Cruz. The coordinates of the five target regions assayed in Figure 3C and 3D are as follows (mm8): chr4:57785004-57785106, chr1:77337105-77337241, chr4:55498595-55498713, chr8:91893769-91893867, chr8:75263369-75263582. The coordinates for the control, nontarget regions are as follows (mm8): chr5:115216446-115216688, chr11:100842467-100842708, chr14:88596378-88596635.

## Supporting Information

Figure S1 (A) CHD7 ChIP in wild type and *Chd7* null ES cells. (B) CoIP experiments.
(1.55 MB EPS)

Figure S2 CHD7 ChIP-seq signals at genes that mediate ES cell self-renewal and pluripotency.
(1.23 MB EPS)

Figure S3 *Chd7* null ES cells differentiate normally into embryoid bodies. qRT-PCR of indicated genes in one wild type and two *Chd7* null ES cells before, during, and after differentiation into embryoid bodies. Other genes quantified include *Sox1*, *Fgf5*, *Gsc*, *T (brachyury)*, *Afp*, *Sox17*, *Gata6*, *Sox7*, and *Hhex* (not shown). The expression of these genes in two *Chd7 ^−/−^* ES cell lines was not significantly different from that in wildtype ES cells.
(1.28 MB EPS)

Figure S4 CHD7 transcriptional regulation is dosage-dependent. (a) (left) Distance versus the average number of CHD7 binding sites per gene for genes that decrease (red), increase (green), or remain the same (black) in *Chd7* heterozygotes as compared to wild type cells. Similar to the comparisons between wild type and null cells, CHD7 sites correlate best with genes that increase in expression upon reduction of CHD7 levels. The distribution of expression of genes in each gene set are shown on the right (b) Same analysis as in (a), but comparing *Chd7* heterozygous cells to *Chd7* homozygous null ES cells.
(0.96 MB EPS)

Figure S5 Absence of CHD7 does not affect reprogramming. Wild type and *Chd7* null ES cells were fused with human B lymphocytes to create heterokaryons. Expression of the indicated genes was measured by qRT-PCR before cell fusion (hB), immediately after cell fusion (d0), and each day following for 3 days. *hHprt* served as a control gene marker. Yellow, green, and blue represent relative expression levels measured in wild type and two independent *Chd7* null ES cell lines, respectively.
(0.54 MB EPS)

Table S1 Genes that decrease upon loss of CHD7.
(0.09 MB XLS)

Table S2 Genes that increase upon loss of CHD7.
(0.13 MB XLS)

Table S3 Genes that are non-differentially expressed.
(0.11 MB XLS)

Table S4 CHD7 sites tested for enhancer activity.
(0.03 MB XLS)
